# Correlation analysis between occupational stress and metabolic syndrome in workers of a petrochemical enterprise: based on two assessment models of occupational stress

**DOI:** 10.1186/s12889-024-18305-3

**Published:** 2024-03-14

**Authors:** Min Zhang, Bin Liu, Weiyi Ke, Yashi Cai, Lingyu Zhang, Weixu Huang, Xuehua Yan, Huifeng Chen

**Affiliations:** 1 Guangdong Province Hospital for Occupational Disease Prevention and Treatment, 510300 Guangzhou, Guangdong China; 2 School of Public Health, Shanxi Medical University, 030001 Taiyuan, Shanxi China; 3 Shenzhen Luohu People’s Hospital, 518000 Shenzhen, Guangdong China

**Keywords:** Occupational stress, Job demand-control, Effort-reward imbalance, Metabolic syndrome, Petrochemical enterprise

## Abstract

**Background:**

Occupational stress is becoming a common phenomenon around the world. Being in a high occupational stress state for a long time may destroy the metabolic balance of the body, thereby increasing the risk of metabolic diseases. There is limited evidence regarding the correlation between occupational stress and metabolic syndrome (MetS), particularly in the petrochemical workers.

**Methods:**

A total of 1683 workers of a petrochemical enterprise in China were included in the survey by cluster sampling method. The occupational stress assessment was carried out by the Job Content Questionnaire and the Effort-Reward Imbalance Questionnaire, and the general demographic characteristics, work characteristics, occupational hazards, lifestyle and health examination data of the participants were collected. Logistic regression and multiple linear regression were used to analyze the correlations and influencing factors between occupational stress and its dimensions with MetS and its components.

**Results:**

A total of 1683 questionnaires were sent out, and 1608 were effectively collected, with an effective recovery rate of 95.54%. The detection rates of occupational stress in Job Demand-Control (JDC) and Effort-Reward Imbalance (ERI) models were 28.4% and 27.2%, respectively. In this study, 257 participants (16.0%) were diagnosed with MetS. Compared with the non-MetS group, body mass index (BMI), waist circumference (WC), systolic blood pressure (SBP), diastolic blood pressure (DBP), triglycerides (TG) and fasting blood-glucose (FBG) levels were significantly higher in the MetS group, and high density lipoprotein-cholesterol (HDL-C) levels were significantly lower (*P* < 0.001). The results of multiple linear regression showed that after adjusting for nation, marital status, education, work system, smoking and drinking, and further adjusting for occupational hazards, the D/C ratio was significantly negatively correlated with SBP in the JDC model. Social support was negatively correlated with WC. In the ERI model, there was a significant positive correlation between over-commitment and FBG.

**Conclusions:**

The detection rates of occupational stress and MetS were high in workers of a petrochemical enterprise. In the JDC model, occupational stress was negatively correlated with SBP, and social support was negatively correlated with WC. In the ERI model, there was a significantly positive correlation between over-commitment and FBG.

**Supplementary Information:**

The online version contains supplementary material available at 10.1186/s12889-024-18305-3.

## Background

In recent years, the economic and health burdens caused by occupational stress have become a global public health research hotspot. Occupational stress and its health impacts on occupational populations require urgent attention [1, 2]. Globally, it has been reported that about 3 million workers are exposed to occupational stress, and the prevalence of occupational stress ranges from 30 to 52.5% [3]. Occupational stress generally refers to the process by which psychological experiences and work demands produce short-term or long-term changes in physical and mental health [4]. Long-term occupational stress can lead to various physical and mental diseases, including cardiovascular diseases [5], musculoskeletal disorders [6], anxiety [7], burnout [8], etc., which reduce job satisfaction and work efficiency [9], and increase the tendency for resignation [10] and the incidence of occupational injury [11]. Some assessment models have been validated to be used to assess the potential association between occupational stress, health and diseases, including the Job Demand-Control (JDC) model and the Effort-Reward Imbalance (ERI) model [12]. The JDC model of occupational stress is generated by the interaction between high demand and low control, and is used to measure job demand (time pressure, task complexity, etc.) and job control (decision-making freedom, etc.). The ERI model of occupational stress is generated by the interaction between high effort and low reward [13], which is used to measure the perceived reward (including salary, promotion opportunities, etc.) and the perceived effort (such as job requirements, etc.) of employees. It has been shown that occupational stress, as assessed using both models, is associated with poor physical and mental health outcomes [14, 15].

Metabolic syndrome (MetS) is caused by a combination of cardiovascular risk factors including insulin resistance, obesity, dyslipidemia and hypertension [16]. The main clinical manifestations were atherosclerosis, tissue fibrosis [17] and abdominal obesity [18], etc. Studies have shown that the prevalence of MetS is 20–30% in the adult population in most countries [19], and deaths from MetS account for two-thirds of deaths from non-communicable chronic diseases [20]. In addition, MetS increases the risk of cancer and cardiovascular disease [21, 22], contributes to the development of chronic kidney disease [23], and can have an impact on cognitive function [24]. A meta-analysis involving different occupational groups such as police, nurses and workers revealed evidence of an association between occupational stress and an increased risk of MetS [25]. In addition, the association between occupational stress and MetS components has been confirmed by many studies [26, 27]. At present, most studies have focused on occupational groups such as police and medical staff, and the health effects of various occupational stress models on different occupational groups are different. In addition, some unadjusted confounding factors in the above studies may also lead to confounding bias. Therefore, in different occupational stress assessment models, the correlation between occupational stress and MetS and its components in different occupational populations needs to be explored urgently.

As an important pillar industry of the national economy, petrochemical enterprises play a key role in meeting the growing demand of Chinese economy. However, as a special occupational group, workers in petrochemical enterprises have more serious occupational health and safety problems such as toxic and harmful substances, high temperature and high pressure due to the particularity of production technology and raw and auxiliary materials. In addition to long-term exposure to chemical poisons, noise, dust, ionizing radiation and other occupational harmful factors [28], workers in petrochemical enterprises are also exposed to a variety of stressors such as night shifts [29], which increase the risk of occupational stress. A cohort study of Korean workers showed that long-term exposure to occupational hazards such as physical factors, chemical toxicants and dusts can lead to occupational stress in workers and have adverse effects on their physical and mental health [30]. At the same time, with the rapid development of social economy and the transformation of industrialization mode, workers in petrochemical enterprises need to constantly receive training in new knowledge and technology, which brings additional pressure to their physical and mental health [31]. At present, there is a lack of systematic studies on the health effects of occupational stress on petrochemical workers. Therefore, it is necessary to investigate the correlation between occupational stress and MetS among workers in petrochemical enterprises.

Based on this, this study used a cross-sectional survey, combined with JDC and ERI assessment models, combined with the unique occupational hazards, individual characteristics, and working characteristics to investigate the occupational stress rate and MetS detection rate of workers in a petrochemical enterprise in China, to analyze the correlation between occupational stress and MetS and its components and to explore the influencing factors of MetS and its components among workers in a petrochemical enterprise. The aim of this study is to provide an important scientific basis for the early intervention of occupational stress and the improvement of physical and mental health of workers in petrochemical enterprises, which is of great significance for alleviating occupational stress of workers in petrochemical enterprises, preventing and controlling MetS, promoting the construction of healthy enterprises, and improving the quality of professional life of workers.

## Methods

### Participants

The workers of a petrochemical enterprise in China were selected as the survey participants by the cluster sampling method. Inclusion criteria included age ≥ 18 years, working years ≥ 1 year, no diagnosis of mental disorders, and no family history of mental disorders. In accordance with the Declaration of Helsinki, informed consent was obtained from all study participants after they were informed of the details of the study before their participations. The study was reviewed and approved by the Medical Ethics Committee of Guangdong Province Hospital for Occupational Disease Prevention and Treatment (No. GDHOD MEC 2018011).

### The demographic questionnaire

The self-designed basic information questionnaire was used for face-to-face investigation. Basic information such as individual characteristics (gender, age, nation, marital status, education), working characteristics (working years, working system, monthly income), occupational hazards (chemical toxicant, noise, dust, high temperature, video display terminal (VDT) operation, electromagnetic radiation, ionizing radiation) and lifestyle (smoking, drinking) were obtained. Among them, smoking is defined as cumulative smoking for more than 6 months [32], drinking is defined as consuming any amount of alcohol at least twice in the past year [33].

### Occupational stress survey

The Job Content Questionnaire (JCQ) and the Effort-Reward Imbalance Questionnaire (ERIQ) were adopted to assess the occupational stress of the participants. Both questionnaires used a likert 4-point scale, rated from 1 to 4, from “disagree entirely” to “agree entirely”. The JCQ includes 22 items in three dimensions: job demand (5 items), job control (9 items) and social support (8 items). The Cronbach’s α coefficient of the questionnaire was 0.853 and the KMO value was 0.900. D/C ratio = job demand/(job control ×5/9) [34], D/C ratio > 1 means a high level of occupational stress. The ERIQ includes three dimensions of effort (6 items), reward (11 items) and over-commitment (6 items), with a total of 23 items. The Cronbach’s α coefficient of the questionnaire was 0.720 and the KMO value was 0.890. E/R ratio = effort/(reward ×6/11) [35], E/R ratio > 1 means a high level of occupational stress.

### Physical and biochemical measurements

Standard methods [36] were used to measure height, weight and waist circumference (WC). The height was measured by a metal column altimeter, the participants were barefooted and stood at attention on the base of the altimeter, with their heels, sacrum and scapula between the pillars close to the altimeter. The standing height was measured with an accuracy of 0.1 cm. Weight was measured using a double-scale lever weight scale with an accuracy of 0.1 kg and the estimated weight of the clothing was subtracted (0.5 kg in summer; 1.0 kg in spring/autumn; 2.0-2.5 kg in winter). Waist circumference was measured using a soft ruler at the midpoint between the lowest rib edge and the iliac crest with an accuracy of 0.1 cm. The body mass index (BMI) was calculated from the measurements. Systolic blood pressure (SBP) and diastolic blood pressure (DBP) were measured using a standard mercury sphygmomanometer. The participants avoided strenuous exercise and rested for at least 5 min before the measurement. The right upper arm of the participant was fully exposed in the sitting position, and the blood pressure in the brachial artery of the right upper arm was measured by experienced staff using an electronic sphygmomanometer that had been qualified for measurement. Before the investigation, the participants fasted for at least 12 h, about 5 ml peripheral venous blood was collected from the participants, the collected blood samples were centrifuged at 3000r/min for 10 min on a horizontal centrifuge, and the serum was collected and used by an automatic biochemical analyzer (Mindray-2000, BS-2000). Fasting blood-glucose (FBG) was measured by glucose oxidase method, and Triglycerides (TG) was measured by oxidase method. High density lipoprotein-cholesterol (HDL-C) was measured by direct method.

### Diagnostic criteria of MetS

According to the CDS-2016 Diagnostic Criteria for Metabolic Syndrome recommended by the China Adult Blood Lipid Abnormality Prevention Guide (2016), those with the following 3 or more items were assessed as having MetS:

1. Central obesity and (or) abdominal obesity: WC ≥ 90 cm for men and ≥ 85 cm for women;
2. Hyperglycemia: FBG ≥ 6.10 mmol/L or 2 h after glucose loading blood sugar ≥ 7.80 mmol/L and (or) have been diagnosed with diabetes and treated;
3. Hypertension: blood pressure ≥ 130/85 mmHg(1 mmHg = 0.133 kPa) and (or) have been diagnosed with hypertension and treated;
4. Fasting TG ≥ 1.7 mmol/L;
5. Fasting HDL-C < 1.0mmol /L.

### Quality control

The questionnaire survey was completed by trained investigators. Before the survey, the purpose of the study and the content of the questionnaire were explained to the participants in detail, and the basic information of the participants was collected by face to face interview. The questionnaires were reviewed after collection, and the participants with missing health examination data and missing items in the questionnaire were excluded. Data were entered by double entry.

### Statistical analysis

The Epidata 3.1 software was utilized for questionnaire data entry, while the statistical analysis was conducted using SPSS 25.0 software. Descriptive statistics were employed to present measurement data as mean ± standard deviation $(\bar x \pm s)$. A T-test was performed to compare means between the two groups. Enumeration data were described using rates or component ratios, and comparisons between groups were made using Pearson χ² test, trend χ² test, or Fisher’s exact test. Each dimension of occupational stress in both models was categorized into high-level and low-level groups based on the median value, and independent sample T-tests were used to compare the levels of MetS components in each group. Logistic regression analysis was applied to identify influencing factors of MetS in both occupational stress models. Multiple linear regression analysis was conducted to determine influencing factors associated with different components of MetS in 

both occupational stress models. The significance level for all tests was set at α = 0.05 (two-tailed).

## Results

### Basic characteristics of participants

A total of 1683 questionnaires were distributed in this survey, and 1608 were effectively collected, with an effective recovery rate of 95.54%. Among the 1608 participants included in this study, 1402 (87.2%) were males and 206 (12.8%) were females. The mean age was 36.73 ± 8.74 years, and the mean working years was 14.51 ± 9.11 years. Most of them were aged 30–39 (37.3%), 1307 were married (81.3%), 856 were undergraduate or above (53.2%), 870 were shift workers (54.1%), 948 were exposed to chemical toxicant(59.0%), 1212 were exposed to noise (75.4%), and 801 were exposed to dust (49.8%). Among them, 27.5% smoked and 62.0% drank alcohol (Table 1).

**Table 1:** Results of MetS detection rate in participants of different individual characterization groups

Characteristics	Total (*n* = 1608)	MetS (*n* = 257)	Non-MetS (*n* = 1351)	*P* value
**Mean ± SD**				
BMI (kg/m^2^)	24.10 ± 2.97	26.87 ± 2.82	23.57 ± 2.70	< 0.001
WC (cm)	85.50 ± 8.67	94.42 ± 6.90	83.80 ± 7.90	< 0.001
DBP (mmHg)	81.50 ± 9.24	88.81 ± 9.20	80.11 ± 8.57	< 0.001
SBP (mmHg)	121.60 ± 12.77	130.74 ± 13.28	119.87 ± 11.91	< 0.001
TG (mmol/L)	1.58 ± 1.14	3.03 ± 1.67	1.30 ± 0.74	< 0.001
HDL-C (mmol/L)	1.34 ± 0.31	1.07 ± 0.22	1.38 ± 0.30	< 0.001
FBG (mmol/L)	4.49 ± 1.01	5.10 ± 1.78	4.38 ± 0.72	< 0.001
**N (%)**				
Gender				
Male	1402(87.2%)	252(98.1%)	1150(85.1%)	< 0.001
Female	206(12.8%)	5(1.9%)	201(14.9%)	
Age(years)				
20–29	439(27.3%)	41(16.0%)	398(29.5%)	< 0.001^a^
30–39	599(37.3%)	96(37.4%)	503(37.2%)	
40–49	399(24.8%)	80(31.1%)	319(23.6%)	
≥ 50	171(10.6%)	40(15.6%)	131(9.7%)	
Nation				
Han	1542(95.9%)	243(94.6%)	1299(96.2%)	0.236
Minority	66(4.1%)	14(5.4%)	52(3.8%)	
Marital status				
Single	301(18.7%)	27(10.5%)	274(20.3%)	< 0.001
Married	1307(81.3%)	230(89.5%)	1077(79.7%)	
Education				
Junior college and below	752(46.8%)	139(54.1%)	613(45.4%)	0.01
Undergraduate or above	856(53.2%)	118(45.9%)	738(54.6%)	
Working years(years)				
≤ 10	734(45.6%)	80(31.1%)	654(48.4%)	< 0.001^a^
11–20	425(26.4%)	75(29.2%)	350(25.9%)	
> 20	449(27.9%)	102(39.7%)	347(25.7%)	
Work system				
Regular day shift	738(45.9%)	93(36.2%)	645(47.7%)	0.001
Shift work	870(54.1%)	164(63.8%)	706(52.3%)	
Chemical toxicant				
Yes	948(59.0%)	158(61.5%)	790(58.5%)	0.370
No	660(41.0%)	99(38.5%)	561(41.5%)	
Noise				
Yes	1212(75.4%)	211(82.1%)	1001(74.1%)	0.006
No	396(24.6%)	46(17.9%)	350(25.9%)	
Dust				
Yes	801(49.8%)	144(56.0%)	657(48.6%)	0.030
No	807(50.2%)	113(44.0%)	694(51.4%)	
High temperature				
Yes	855(53.2%)	146(56.8%)	709(52.5%)	0.202
No	753(46.8%)	111(43.2%)	642(47.5%)	
VDT operation				
Yes	240(14.9%)	41(16.0%)	199(14.7%)	0.614
No	1368(85.1%)	216(84.0%)	1152(85.3%)	
Electromagnetic radiation				
Yes	128(8.0%)	22(8.6%)	106(7.8%)	0.698
No	1480(92.0%)	235(91.4%)	1245(92.2%)	
Ionizing radiation				
Yes	253(15.7%)	43(16.7%)	210(15.5%)	0.632
No	1355(84.3%)	214(83.3%)	1141(84.5%)	
Monthly income (yuan)				
< 10,000	430(26.7%)	44(17.1%)	386(28.6%)	< 0.001^a^
10,000–12,000	486(30.2%)	74(28.8%)	412(30.5%)	
> 12,000	692(43.0%)	139(54.1%)	553(40.9%)	
Smoking				
Yes	443(27.5%)	105(40.9%)	338(25.0%)	< 0.001
No	1165(72.5%)	152(59.1%)	1013(75.0%)	
Drinking				
Yes	997(62.0%)	178(69.3%)	819(60.6%)	0.009
No	611(38.0%)	79(30.7%)	532(39.4%)	

Note: ^a^*P* values were calculated from trend χ² Test

### The detection rate of MetS in different individual characteristic groups of workers in a petrochemical enterprise

The detection rate of MetS among the participants was 16.0% (257/1608). There were statistically significant differences in MetS detection rates among different gender, age, marital status, education, working years, work system, noise, dust, monthly income, smoking and drinking groups (*P* < 0.05). Compared with the non-MetS group, the levels of BMI, WC, SBP, DBP, TG and FBG were significantly higher in the MetS group (*P* < 0.001), and the levels of HDL-C were significantly lower in the MetS group (*P* < 0.001) (Table 1).

### Comparison of components of MetS in two occupational stress models and each dimension level

The results showed that in JDC and ERI models, the detection rates of occupational stress in a petrochemical enterprise were 28.4% and 27.2%, respectively. In the JDC model, SBP level in D/C ratio > 1 group was significantly decreased compared with that in D/C ratio ≤ 1 group (*P* < 0.05). Compared with low social support group, SBP and DBP levels in high social support group were significantly increased (*P* < 0.05). In the ERI model, compared with E/R ratio ≤ 1 group, HDL-C level in E/R ratio > 1 group was significantly decreased, and WC and TG levels were significantly increased in E/R ratio > 1 group (*P* < 0.05). HDL-C levels were significantly lower and TG levels were significantly higher in the high effort group compared to the low effort group (*P* < 0.05) (Table 2).

**Table 2:** Comparison of component levels of MetS in different occupational stress and dimensional level groups

	SBP(mmHg)	DBP(mmHg)	FBG(mmol/L)	HDL-C(mmol/L)	WC(cm)	TG(mmol/L)
JDC model						
D/C						
> 1	120.36 ± 12.45^*^	80.84 ± 9.34	4.45 ± 0.98	1.35 ± 0.32	85.53 ± 8.81	1.59 ± 1.19
≤ 1	122.10 ± 12.87	81.76 ± 9.19	4.51 ± 1.02	1.33 ± 0.31	85.48 ± 8.62	1.57 ± 1.12
*t* value	2.462	1.802	1.118	-0.927	-0.101	-0.251
*P* value	0.014	0.072	0.264	0.354	0.920	0.802
job demand						
high	121.06 ± 12.41	81.14 ± 9.15	4.49 ± 0.94	1.35 ± 0.31	85.13 ± 8.54	1.58 ± 1.14
low	122.01 ± 13.03	81.76 ± 9.30	4.50 ± 1.05	1.33 ± 0.31	85.77 ± 8.76	1.58 ± 1.14
*t* value	1.468	1.327	0.195	-1.291	1.456	0.079
*P* value	0.142	0.185	0.845	0.197	0.146	0.937
job control						
high	122.18 ± 13.16	81.64 ± 9.54	4.50 ± 0.90	1.35 ± 0.30	84.99 ± 8.25	1.57 ± 1.16
low	121.30 ± 12.56	81.43 ± 9.07	4.49 ± 1.06	1.33 ± 0.31	85.76 ± 8.88	1.59 ± 1.13
*t* value	-1.324	-0.438	-0.288	-1.006	1.686	0.356
*P* value	0.186	0.662	0.774	0.315	0.092	0.722
social support						
high	122.83 ± 13.21^*^	82.28 ± 9.66^*^	4.49 ± 1.06	1.33 ± 0.28	85.20 ± 9.27	1.59 ± 1.18
low	121.17 ± 12.59	81.23 ± 9.07	4.49 ± 0.99	1.34 ± 0.32	85.60 ± 8.45	1.58 ± 1.12
*t* value	-2.285	-2.013	0.166	0.215	0.765	-0.158
*P* value	0.022	0.044	0.868	0.830	0.445	0.874
ERI model						
E/R ratio						
> 1	121.91 ± 11.91	81.84 ± 9.26	4.52 ± 1.18	1.31 ± 0.31^*^	86.54 ± 8.54^*^	1.71 ± 1.32^*^
≤ 1	121.49 ± 13.09	81.38 ± 9.23	4.48 ± 0.93	1.35 ± 0.31	85.10 ± 8.69	1.53 ± 1.06
*t* value	-0.620	-0.890	-0.686	2.127	-2.967	-2.614
*P* value	0.535	0.374	0.493	0.034	0.003	0.009
effort						
high	121.35 ± 12.42	81.41 ± 9.16	4.49 ± 1.09	1.32 ± 0.30^*^	85.73 ± 8.47	1.65 ± 1.23^*^
low	121.81 ± 13.05	81.57 ± 9.30	4.49 ± 0.93	1.35 ± 0.31	85.30 ± 8.83	1.52 ± 1.06
*t* value	0.724	0.345	-0.030	2.146	-0.987	-2.302
*P* value	0.469	0.730	0.976	0.032	0.324	0.021
reward						
high	121.85 ± 12.64	81.91 ± 9.16	4.49 ± 0.85	1.33 ± 0.29	85.38 ± 9.08	1.59 ± 1.14
low	121.48 ± 152.85	81.29 ± 9.27	4.49 ± 1.08	1.34 ± 0.32	85.55 ± 8.45	1.57 ± 1.14
*t* value	-0.551	-1.287	0.100	0.424	0.368	-0.323
*P* value	0.582	0.198	0.920	0.672	0.713	0.747
Over-commitment						
high	121.59 ± 12.24	81.45 ± 9.06	4.51 ± 1.14	1.32 ± 0.31	85.55 ± 8.44	1.64 ± 1.19
low	121.61 ± 13.10	81.54 ± 9.35	4.48 ± 0.91	1.34 ± 0.31	85.46 ± 8.82	1.54 ± 1.11
*t* value	0.041	0.188	-0.709	1.270	-0.210	-1.768
*P* value	0.947	0.851	0.478	0.204	0.834	0.077

### Logistic regression analysis of the correlation between occupational stress and MetS in two occupational stress models among workers in a petrochemical enterprise

**Fig. 1:**
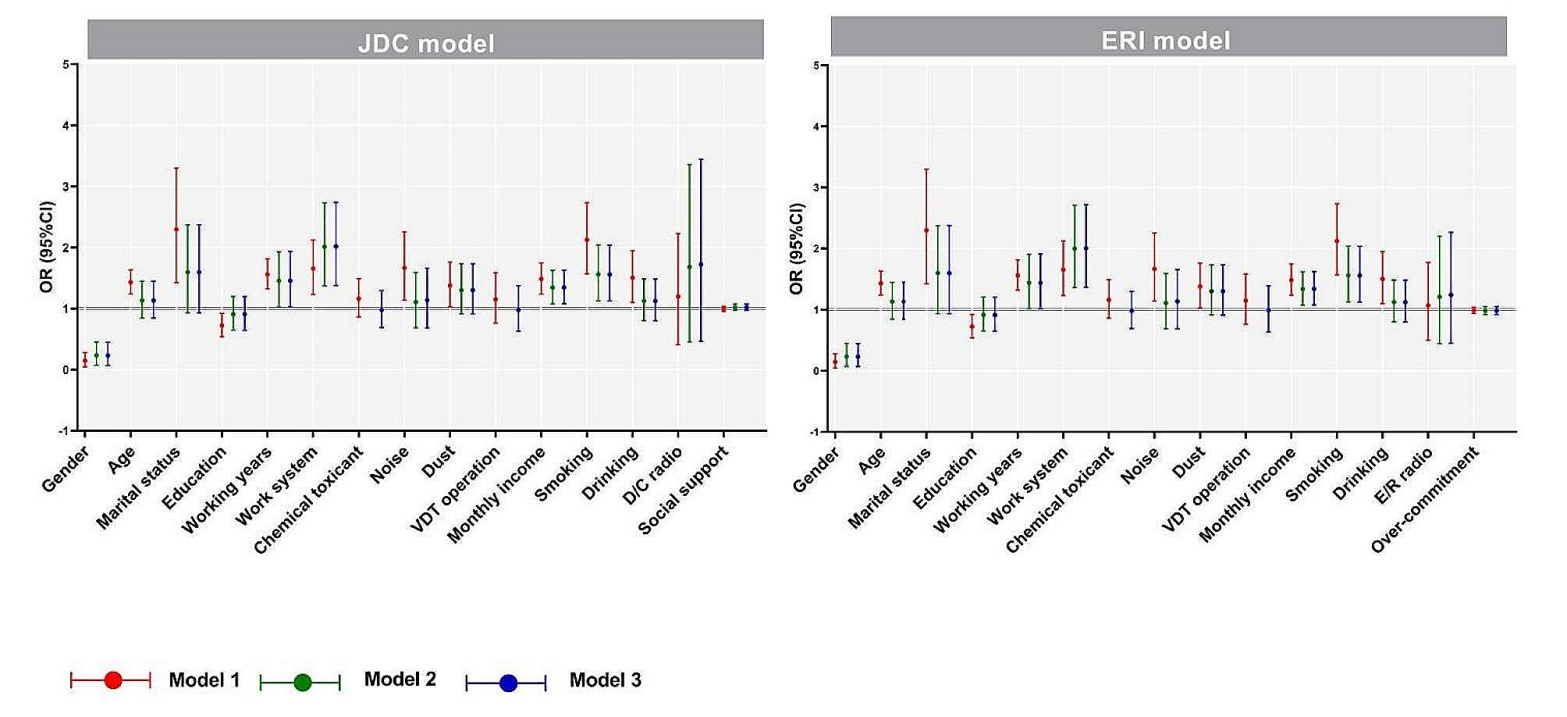
Logistic regression analysis of MetS influencing factors of participants in JDC and ERI models Note: Gender was “male” as the reference group; Age was “<30 years old” as the reference group; Marital status was “single” as the reference group; Education was “junior college and below” as the reference group; Working system was “regular day shift” as the reference group; Chemical toxicant, noise, dust and VDT operation were “no” as the reference group; Monthly income was “<10000 yuan” as the reference group; Smoking and drinking were “no” as the reference group. D/C ratio, social support, E/R ratio and over-commitment are continuous variables Model 1: Unadjusted crude model; Model 2: Adjusted for variables with *p* < 0.05 in Model 1; Model 3: Based on model 2, with adjustments for chemical toxicant and VDT operation

The results showed that in model 2, after adjusting for independent variables *P* < 0.05 in model 1, and model 3, after further adjusting for Chemical toxicant and VDT operation, neither D/C ratio nor social support were significantly associated with MetS in the JDC model (*P* > 0.05). In the ERI model, E/R ratio and over-commitment were not significantly correlated with MetS (*P* > 0.05) (Fig. 1).

### Multiple linear regression analysis of occupational stress, its dimensions and its correlation with MetS components in a petrochemical enterprise in two occupational stress models

**Fig. 2:**
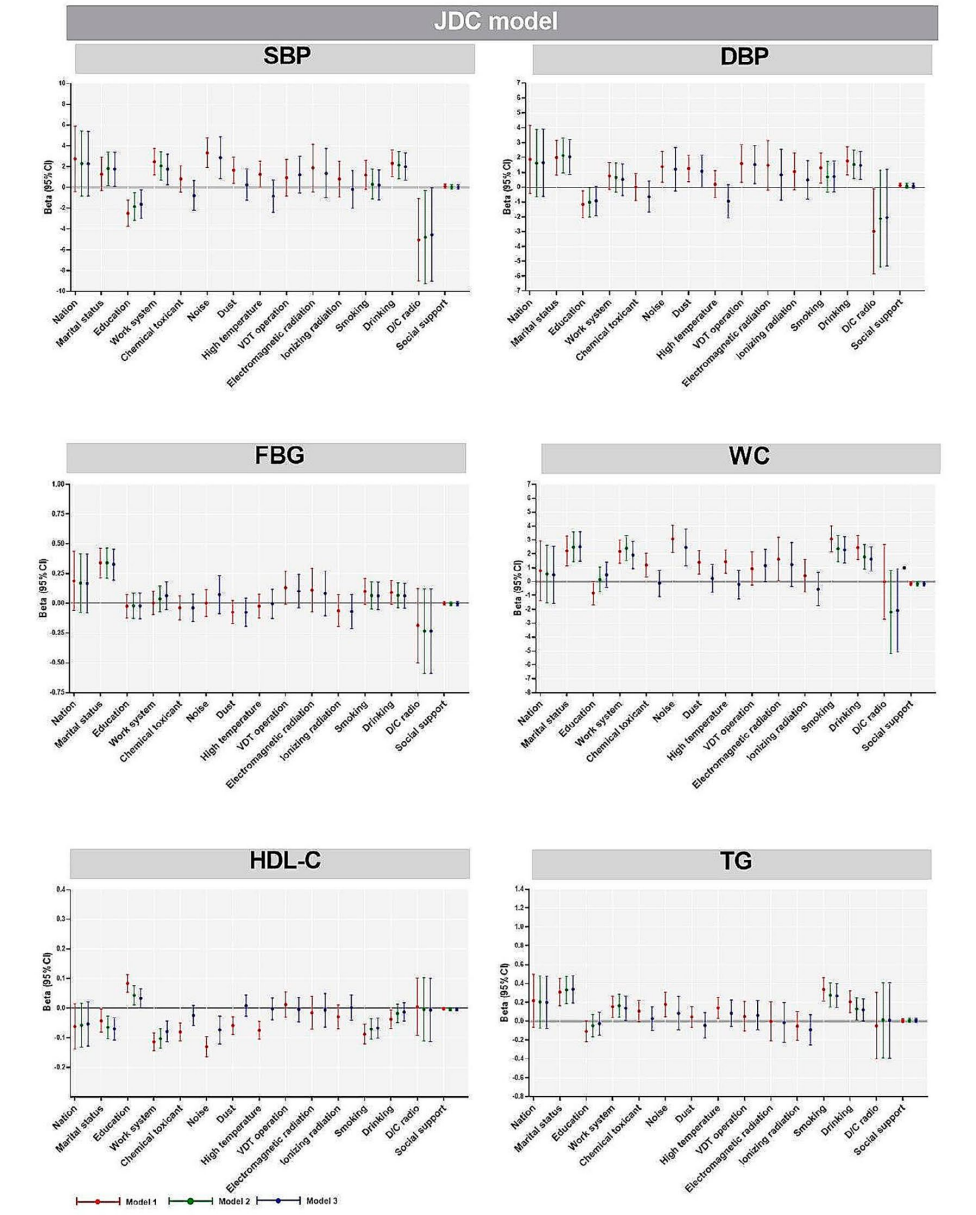
Multiple linear regression analysis of factors influencing participants’ MetS components in the JDC model Note: Nation was “han” as the reference group; Marital status was “single” as the reference group; Education was “junior college and below” as the reference group; Working system was “regular day shift” as the reference group; Chemical toxicant, noise, dust, high temperature, VDT operation, electromagnetic radiation and ionizing radiation were “no” as the reference group; Smoking and drinking were “no” as the reference group. D/C ratio and social support are continuous variables Model 1: Unadjusted crude model; Model 2: Based on model 1, with adjustments for nation, marital status, education, work system, smoking and drinking; Model 3: Based on model 2, with adjustments for occupational hazards (chemical toxicant, noise, dust, high temperature, VDT operation, electromagnetic radiation and ionizing radiation)

**Fig. 3:**
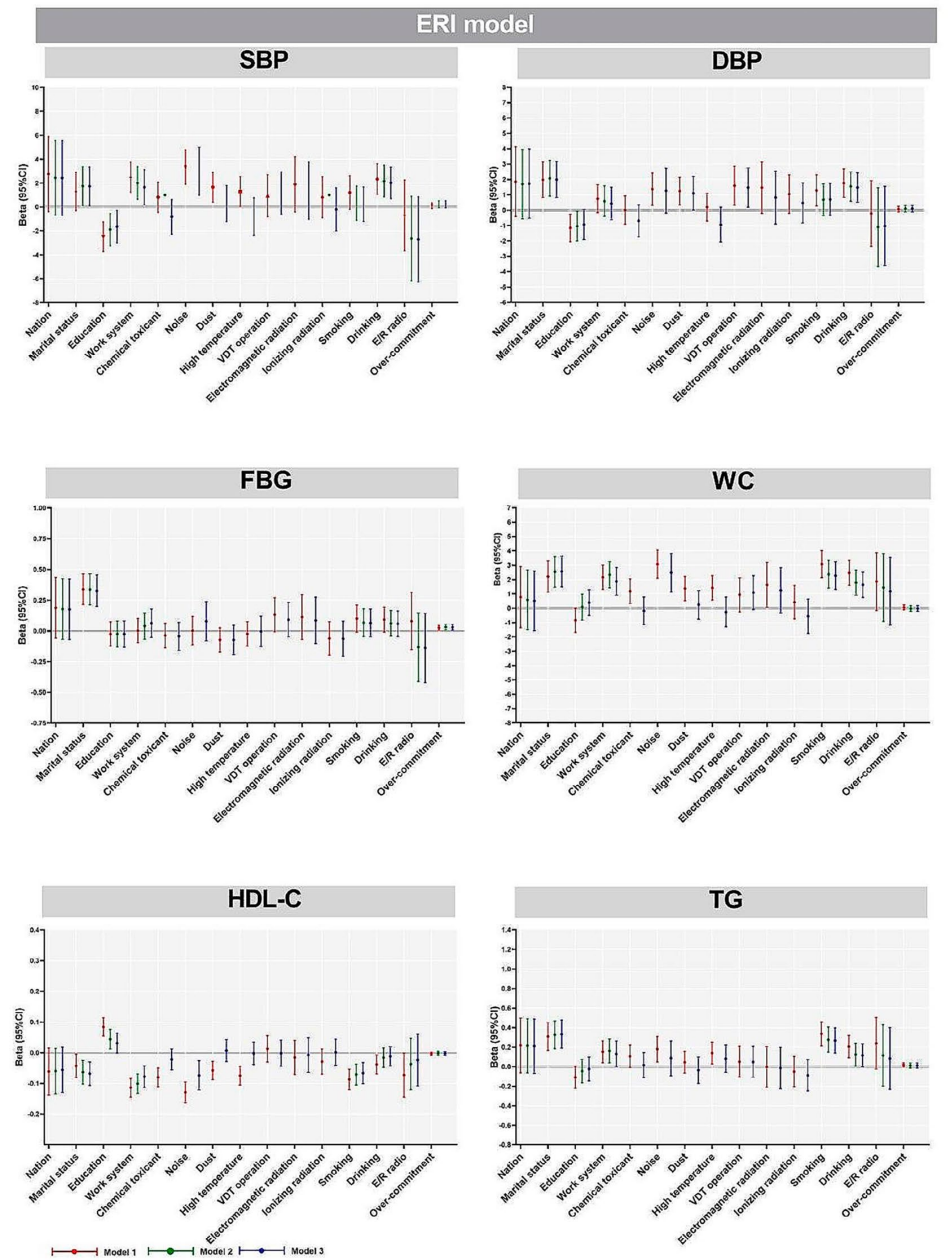
Multiple linear regression analysis of factors influencing participants’ MetS components in the ERI model Note: Nation was “han” as the reference group; Marital status was “single” as the reference group; Education was “junior college and below” as the reference group; Working system was “regular day shift” as the reference group; Chemical toxicant, noise, dust, high temperature, VDT operation, electromagnetic radiation and ionizing radiation were “no” as the reference group; Smoking and drinking were “no” as the reference group. E/R ratio and over-commitment are continuous variables Model 1: Unadjusted crude model; Model 2: Based on model 1, with adjustments for nation, marital status, education, work system, smoking and drinking; Model 3: Based on model 2, with adjustments for occupational hazards (chemical toxicant, noise, dust, high temperature, VDT operation, electromagnetic radiation and ionizing radiation)

The results showed that in model 3, after adjusting for nation, marital status, education, work system, smoking, drinking and occupational hazards (chemical toxicant, noise, dust, high temperature, VDT operation, electromagnetic radiation and ionizing radiation), D/C ratio was significantly negatively correlated with SBP in JDC model (*P* < 0.05); Social support was negatively correlated with WC (*P* < 0.05). In the ERI model, there was a significant positive correlation between over-commitment and FBG (*P* < 0.05) (Figures 2 and 3).

Married and high SBP (*P* < 0.05), high DBP (*P* < 0.01), high FBG (*P* < 0.001), high WC (*P* < 0.001), low HDL-C (*P* < 0.001), high TG (*P* < 0.001) were significantly correlated. Higher education and lower SBP (*P* < 0.05) and high HDL-C (*P* < 0.05) were significantly correlated. Shift work was associated with high SBP (*P* < 0.05), high WC (*P* < 0.001), low HDL-C (*P* < 0.001), high TG (*P* < 0.05) were significantly correlated. Smoking and high WC (*P* < 0.001), low HDL-C (*P* < 0.001), high TG (*P* < 0.001) were significantly correlated. Drinking and high SBP (*P* < 0.011), high DBP (*P* < 0.01), high WC (*P* < 0.001) and high TG (*P* < 0.05) were significantly correlated. Noise and high SBP (*P* < 0.01), high WC (*P* < 0.001), low HDL-C (*P* < 0.01) were significantly correlated. Dust exposure was significantly associated with high DBP (*P* < 0.05). VDT operation was significantly associated with high DBP (*P* < 0.05) (Figures 2 and 3, Table S1).

## Discussion

This study investigated the current status of occupational stress and MetS among workers of a petrochemical enterprise in China, and analyzed the correlation between occupational stress and MetS and its components. The results showed that there was no correlation between occupational stress and MetS in the two models, but there was a certain correlation with MetS components. In the JDC model, occupational stress was negatively correlated with SBP, and social support was negatively correlated with WC. In the ERI model, over-commitment was positively correlated with FBG.

With the transformation from traditional biomedical model to bio-psycho-social medical model, more and more researches focus on occupational psychosocial factors [37]. Occupational stress has been widely concerned as a psychosocial risk factor [26]. At the same time, MetS, as a collection of a series of cardiovascular risk factors, can lead to an increased risk of type II diabetes, cardiovascular disease, and other chronic diseases [38]. Early screening of MetS and research on its influencing factors are of great practical significance for early intervention, prevention and control of chronic diseases. Petrochemical enterprise workers, as a special occupational group, face a large workload and long-term exposure to a variety of occupational hazards in the production line. In addition, they are also faced with a variety of stressors such as irregular work and rest caused by shift work and night shift work, which make workers prone to adverse work emotions in the process of production and cause occupational stress, which has a significant impact on physical and mental health. Therefore, this study combined two occupational stress assessment models to conduct a comprehensive and systematic assessment of occupational stress among workers in a petrochemical enterprise. Not only the correlation between multiple dimensions in the two models and MetS was analyzed, but also the correlation between five components of MetS was analyzed to reveal the correlation between occupational stress and components of MetS.

The results showed that in JDC model and ERI model, the detection rate of high occupational stress of workers in a petrochemical enterprise is 28.4% and 27.2%, respectively. It was slightly higher than the occupational stress detection rate of JDC model reported by Curēus et al. [39] in female shift workers in southern Brazil (24.0%) and the occupational stress detection rate of ERI model reported by Kong et al. [35] in clinical nurses (26.5%), suggesting that there are differences in the occupational stress detection rate of occupational groups in different industries, and the workers in a petrochemical enterprise have high occupational stress. Yan et al. [3] investigated 13,867 industrial workers in China and found that educational level and gender were correlated with occupational stress. Therefore, workers in petrochemical enterprises showed different occupational stress from workers in other industries, and the reasons may be related to sample size, population structure, industry characteristics, and individual characteristics. The detection rate of MetS among workers in a petrochemical enterprise was 16.0%, similar to the results of Rashnuodi et al. [40], which found that 15.1% of petrochemical workers suffered from MetS. This was lower than the MetS detection rate reported by Merces et al. [41] among nursing professionals (24.4%) and Eftekhari et al. [26], among medical university staff (22.1%). The possible reason is that the phenotype of MetS is the result of the interaction of genetic, environmental, and behavioral factors [40]. Therefore, there were differences in the detection rates of MetS among different occupational groups. The above results indicate that there is a high level of occupational stress and a certain degree of prevalence of MetS among workers in a petrochemical enterprise.

In order to exclude the influence of confounding factors, this study firstly excluded participants with a diagnosis of mental disorders and family history of mental disorders. Secondly, multiple linear regression analysis was performed to analyze the correlation between occupational stress and its dimensions and MetS components in the two models after adjusting covariates. The results suggested that there is a significant negative correlation between D/C ratio and SBP in the JDC model, which seems to be inconsistent with the known results. Rosenthal et al. [42] found that the degree of change in blood pressure in daily life may be affected by cultural background and emotional state of cognitive processes and other factors, blood pressure healthy people may be engaged in more occupational stress exposure work, these factors may combine to contribute to the negative association between occupational stress and blood pressure outcomes in workers in petrochemical enterprises. The significant negative correlation between social support and WC level indicated that the degree of central obesity decreased when social support increased, which was consistent with the results of Yoshida et al. [43] and Kshtriya et al. [44], suggesting that social support could alleviate the adverse consequences of occupational stress by regulating the degree of occupational stress. Zeinab et al. [45] also found that higher social support was significantly associated with lower WC in a Canadian elderly cohort study, possibly because social support can bring access to instrumental assistance and information resources, or through emotional or tangible support to buffer the impact of stressful events.

The present study also found that over-commitment was significantly and positively associated with FBG in the ERI model. Irie et al. [46] found that over-commitment was associated with elevated blood glucose levels in manufacturing workers, and the correlation remained after adjusting for covariates, which were analyzed for possible reasons related to cortisol secretion. Allison et al. [47] also found in the study of police officers that occupational stress can lead to increased cortisol secretion, which causes insulin resistance and affects glucose metabolism by promoting hepatic glucongenesis, inhibiting glucose uptake, promoting lipolysis and inhibiting insulin secretion [48]. It is suggested that due to the high job demand and high over-commitment of workers in petrochemical enterprises, the body may affect blood glucose level by regulating cortisol secretion. The two models showed different correlations between occupational stress and MetS components. The possible reason is that the emphasis of JDC model and ERI model is different. According to the theory of JDC model, occupational population have the highest stress when they have high job demand, low job control and low social support [49]. According to the theory of ERI model, occupational population have the highest stress when they effort more in their work but reward less in return [50]. The two models can be combined with different aspects of the social psychological and work environment [51], and the combination of the two models has a good effect on occupational stress assessment [52, 53].

In this study, in the two occupational stress models, SBP, DBP, FBG, WC and TG levels were significantly increased and HDL-C levels were significantly decreased in the married group compared with the single group. The differences in MetS components among different marital status groups may be related to marital quality. Henry et al. [54] found that marital quality affects the occurrence of MetS through depressive symptoms. In addition, several studies have shown that married people are significantly more overweight and obese than single people [55]. Compared with the lower education group, the higher education group had significantly lower SBP and significantly higher HDL-C. In a mendelian randomization study, Howe et al. [56] also found that higher education level can reduce SBP level. A study in the United States also found that higher levels of education were associated with improvements in HDL-C [57]. Compared with the regular day shift group, SBP, WC and TG levels in shift work group were significantly increased, while HDL-C levels were significantly decreased. Khosravipour et al. [58]and Santos et al. [59], both pointed out that shift work system can increase the risk of MetS, possibly because shift work leads to workers’ circadian rhythm disturbance, sleep disorders, irregular eating, etc., resulting in abdominal fat deposition and dyslipidemia. Compared with the non-exposed group, the levels of SBP and WC in the noise-exposed group were significantly increased, and the levels of HDL-C were significantly decreased. In the study, Kupcikova et al. [60], using the UK Biobank database of 502,651 people aged 40–69 years old recruited in the United Kingdom, found that noise exposure can lead to increased levels of cardiovascular disease risk factors such as SBP in the population. Yu et al. [61] showed that as noise exposure increased, the risk of MetS in older MexicAn-American adults gradually increased. The DBP level in the dust-exposed group was significantly higher than that in the non-exposed group. Ishii et al. [62] found a strong correlation between short-term dust-exposure and increased DBP levels. The level of DBP in the VDT operation group was significantly higher than that in the non-VDT operation group. Garcia-Remeseiro et al. [63] had shown that VDT operation workers tend to cause the incidence of musculoskeletal disorders. Another study showed an association between musculoskeletal disorders and the risk of hypertension [64]. It is suggested that VDT operation may have a certain correlation with hypertension. Compared with the non-smoking group, the levels of WC and TG in the smoking group were significantly increased, and the levels of HDL-C were significantly decreased. This is consistent with the results of Efendi et al. [65]. It is suggested that smoking may increase the accumulation of fat and increase insulin resistance, leading to abdominal obesity. Herath et al. [66] pointed out that compared with non-smokers, TG levels in smokers are significantly higher and HDL-C levels are significantly lower. The possible mechanism is that smoking can cause a high concentration of nicotine to enter the blood circulation through the lungs, leading to an increase in free fatty acids in the liver, promoting the production of TG and VLDL-C, and an increase in VLDL-C levels in the blood, contributing to a decrease in HDL-C levels. Compared with the non-drinking group, the levels of SBP, DBP, WC and TG in the drinking group were significantly increased. Xiao et al. [67] found that drinking can cause hypertension and increase WC and TG levels in men. The increase of TG level may be related to alcohol-induced increased secretion of very low density lipoprotein, impaired lipolysis, and increased flow of free fatty acids. Heavy drinkers may be obese due to excessive energy intake, lipid oxidation, and fat accumulation. Another study suggested that drinking may cause blood pressure to increase because it causes blood vessels to constrict, heart rate to increase, sympathetic nervous system activation, and magnesium loss [68].

This study has certain limitations. First, as a cross-sectional study, it is difficult to determine the causal relationship between occupational stress and MetS and its components. Second, only the direct association between occupational stress and MetS and its components was analyzed, and the possible mediating effect of other factors was not considered. In addition, only one petrochemical enterprise in China was selected for the study, and there may be potential confounding factors such as regional characteristics, economic level, dietary habits, and enterprise size, which may affect the universality of the study results. In subsequent studies, cohort studies will be considered to clarify the causal relationship between occupational stress level and MetS, and further path analysis will be conducted to determine whether other factors play a mediating effect on occupational stress and MetS and its components. At the same time, multiple petrochemical enterprises can be combined to carry out multicenter studies to provide important evidence for the study of occupational stress and MetS in petrochemical workers.

## Conclusion

In summary, based on the special occupational group of a petrochemical enterprise, combined with individual characteristics, work characteristics, and lifestyle, this study provides reliable clues for a comprehensive and systematic analysis of the correlation between the two occupational stress patterns and MetS and its components. The results showed that there was no correlation between occupational stress and MetS, but there was a certain correlation between occupational stress and components of MetS. In the JDC model, occupational stress was negatively correlated with SBP, and social support was negatively correlated with WC. In the ERI model, over-commitment was positively correlated with FBG. Our results provide a scientific basis for occupational stress risk assessment and early intervention of MetS for petrochemical workers, and a theoretical basis for government agencies to promote the construction of healthy enterprises. It is recommended that petrochemical enterprises formulate targeted measures for the physical and mental health of employees, provide reasonable rest time for shift workers; strengthen the care of employees, actively carry out team building activities, so as to improve the social support of employees in the working environment. Moreover, engineering protection and personal protection against occupational hazards should be strengthened, and regular physical examinations of workers should be carried out. Petrochemical enterprises should also carry out pre-job training and mental health training activities, encourage employees to relax during non-working hours and reduce their over-commitment levels. Employees should cultivate a good lifestyle, moderate exercise, a balanced diet, and maintain a positive and optimistic attitude.

### Electronic supplementary material

Below is the link to the electronic supplementary material.

Supplementary Material 1

## Data Availability

The data that support the findings of this study are available on request from the corresponding author.

## Abbreviations

JDC: Job demand-control
ERI: Effort-reward imbalance
MetS: Metabolic syndrome
JCQ: Job content questionnaire
ERIQ: Effort-reward imbalance questionnaire
WC: Waist circumference
BMI: Body mass index
SBP: Systolic blood pressure
DBP: Diastolic blood pressure
FBG: Fasting blood-glucose
TG: Triglycerides
HDL-C: High density lipoprotein-cholesterol
VDT: Video display terminals
VLDL-C: Very low density lipoprotein-cholesterol
