# Supplementary material for: Correlation analysis between occupational stress and metabolic syndrome in workers of a petrochemical enterprise: based on two assessment models of occupational stress
Source: BMC Public Health. 2024 Mar 14;24:802. doi: 10.1186/s12889-024-18305-3 (PMC10938751; doi:10.1186/s12889-024-18305-3)
Supplement: Supplementary file 1 — Supplementary Material 1 [file 12889_2024_18305_MOESM1_ESM.docx]

**Supplement Table 1**

*Multiple linear regression analysis of factors influencing participants' MetS components under the JDC model and the ERI model.*

| Variables | SBP | | | DBP | | | FBG | | | WC | | | HDL-C | | | TG | | |
| --- | --- | --- | --- | --- | --- | --- | --- | --- | --- | --- | --- | --- | --- | --- | --- | --- | --- | --- |
|  | β | 95%CI | *P-*value | β | 95%CI | *P-*value | β | 95%CI | *P-*value | β | 95%CI | *P-*value | β | 95%CI | *P-*value | β | 95%CI | *P-*value |
| JDC Model |  |  |  |  |  |  |  |  |  |  |  |  |  |  |  |  |  |  |
| Nation | 2.299 | (-0.818,5.416) | 0.148 | 1.653 | (-0.601,3.908) | 0.151 | 0.167 | (-0.080,0.414) | 0.184 | 0.483 | (-1.587,2.553) | 0.647 | -0.053 | (-0.127,0.021) | 0.159 | 0.200 | (-0.078,0.477) | 0.158 |
| Marital status | 1.771 | (0.153,3.390) | 0.032 | 2.039 | (0.868,3.210) | 0.001 | 0.328 | (0.199,0.456) | ＜0.001 | 2.513 | (1.438,3.588) | ＜0.001 | -0.070 | (-0.108,-0.031) | ＜0.001 | 0.338 | (0.194,0.482) | ＜0.001 |
| Education | -1.606 | (-2.970,-0.242) | 0.021 | -0.928 | (-1.915,0.058) | 0.065 | -0.022 | (-0.130,0.086) | 0.686 | 0.475 | (-0.430,1.381) | 0.304 | 0.033 | (0.001,0.065) | 0.048 | -0.025 | (-0.146,0.097) | 0.687 |
| Work system | 1.755 | (0.291,3.219) | 0.019 | 0.509 | (-0.550,1.568) | 0.346 | 0.064 | (-0.052,0.180) | 0.281 | 1.907 | (0.935,2.879) | ＜0.001 | -0.079 | (-0.114,-0.044) | ＜0.001 | 0.137 | (0.006,0.267) | 0.040 |
| Chemical toxicant | -0.768 | (-2.206,0.669) | 0.295 | -0.647 | (-1.687,0.393) | 0.222 | -0.038 | (-0.152,0.076) | 0.511 | -0.127 | (-1.081,0.828) | 0.7945 | -0.024 | (-0.058,0.010) | 0.163 | 0.026 | (-0.102,0.154) | 0.689 |
| Noise | 2.856 | (0.843,4.869) | 0.005 | 1.203 | (-0.253,2.660) | 0.105 | 0.073 | (-0.087,0.232) | 0.372 | 2.465 | (1.128,3.802) | ＜0.001 | -0.074 | (-0.121,-0.026) | 0.003 | 0.085 | (-0.095,0.264) | 0.355 |
| Dust | 0.266 | (-1.240,1.772) | 0.729 | 1.074 | (-0.015,2.163) | 0.053 | -0.075 | (-0.194,0.044) | 0.218 | 0.233 | (-0.767,1.234) | 0.647 | 0.009 | (-0.027,0.045) | 0.619 | -0.043 | (-0.178,0.091) | 0.525 |
| High temperature | -0.833 | (-2.387,0.721) | 0.293 | -0.949 | (-2.073,0.176) | 0.098 | -0.004 | (-0.127,0.119) | 0.946 | -0.214 | (-1.246,0.818) | 0.685 | -0.003 | (-0.040,0.034) | 0.873 | 0.086 | (-0.053,0.224) | 0.224 |
| VDT operation | 1.236 | (-0.532,3.004) | 0.171 | 1.526 | (0.247,2.805) | 0.019 | 0.103 | (-0.037,0.243) | 0.148 | 1.153 | (-0.021,2.327) | 0.054 | -0.005 | (-0.047,0.037) | 0.820 | 0.064 | (-0.094,0.221) | 0.428 |
| Electromagnetic radiation | 1.365 | (-1.012,3.743) | 0.260 | 0.830 | (-0.890,2.550) | 0.344 | 0.083 | (-0.105,0.272) | 0.385 | 1.222 | (-0.357,2.801) | 0.129 | -0.007 | (-0.063,0.050) | 0.813 | -0.016 | (-0.227,0.196) | 0.885 |
| Ionizing radiation | -0.196 | (-2.000,1.607) | 0.831 | 0.483 | (-0.821,1.788) | 0.468 | -0.069 | (-0.211,0.074) | 0.347 | -0.552 | (-1.750,0.645) | 0.366 | 0.002 | (-0.041,0.045) | 0.928 | 0.002 | (-0.252,0.069) | 0.265 |
| Smoking | 0.240 | (-1.198,1.678) | 0.743 | 0.717 | (-0.323,1.757) | 0.177 | 0.063 | (-0.051,0.177) | 0.276 | 2.276 | (1.321,3.231) | ＜0.001 | -0.067 | (-0.101,-0.033) | ＜0.001 | 0.270 | (0.141,0.398) | ＜0.001 |
| Drinking | 2.010 | (0.692,3.328) | 0.003 | 1.466 | (0.513,2.420) | 0.003 | 0.063 | (-0.041,0.168) | 0.235 | 1.633 | (0.758,2.508) | ＜0.001 | -0.013 | (-0.044,0.018) | 0.421 | 0.119 | (0.002,0.237) | 0.047 |
| D/C radio | -4.547 | (-9.041,-0.052) | 0.047 | -2.056 | (-5.307,1.195) | 0.215 | -0.232 | (-0.588,0.124) | 0.201 | -2.075 | (-5.060,0.910) | 0.173 | -0.007 | (-0.113,0.100) | 0.903 | 0.010 | (-0.391,0.410) | 0.963 |
| Social Support | 0.058 | (-0.173,0.289) | 0.622 | 0.088 | (-0.079,0.255) | 0.299 | -0.004 | (-0.023,0.014) | 0.645 | -0.175 | (-0.328,-0.021) | 0.026 | -0.004 | (-0.009,0.002) | 0.202 | 0.010 | (-0.011,0.030) | 0.346 |
| ERI Model |  |  |  |  |  |  |  |  |  |  |  |  |  |  |  |  |  |  |
| Nation | 2.419 | (-0.702,5.539) | 0.129 | 1.735 | (-0.522,3.993) | 0.132 | 0.177 | (-0.070,0.423) | 0.159 | 0.517 | (-1.555,2.589) | 0.625 | -0.056 | (-0.130,0.018) | 0.139 | 0.210 | (-0.068,0.487) | 0.139 |
| Marital status | 1.725 | (0.106,3.345) | 0.037 | 1.997 | (0.826,3.169) | 0.001 | 0.327 | (0.199,0.455) | ＜0.001 | 2.562 | (1.486,3.637) | ＜0.001 | -0.069 | (-0.107,-0.030) | ＜0.001 | 0.334 | (0.190,0.478) | ＜0.001 |
| Education | -1.638 | (-3.001,-0.276) | 0.018 | -0.934 | (-1.919,0.052) | 0.063 | -0.026 | (-0.133,0.082) | 0.641 | 0.392 | (-0.512,1.297) | 0.395 | 0.032 | (-0.001,0.064) | 0.051 | -0.024 | (-0.145,0.097) | 0.695 |
| Work system | 1.650 | (0.188,3.112) | 0.027 | 0.435 | (-0.623,1.492) | 0.420 | 0.063 | (-0.053,0.178) | 0.286 | 1.864 | (0.893,2.835) | ＜0.001 | -0.078 | (-0.113,-0.043) | ＜0.001 | 0.133 | (0.003,0.263) | 0.045 |
| Chemical toxicant | -0.823 | (-2.265,0.619) | 0.263 | -0.693 | (-1.736,0.350) | 0.193 | -0.044 | (-0.158,0.070) | 0.446 | -0.179 | (-1.137,0.778) | 0.713 | -0.022 | (-0.056,0.012) | 0.207 | 0.018 | (-0.111,0.146) | 0.788 |
| Noise | 2.989 | (0.975,5.003) | 0.004 | 1.274 | (-0.183,2.730) | 0.087 | 0.079 | (-0.080,0.238) | 0.332 | 2.475 | (1.138,3.812) | ＜0.001 | -0.074 | (-0.122,-0.026) | 0.002 | 0.086 | (-0.093,0.265) | 0.345 |
| Dust | 0.285 | (-1.225,1.794) | 0.711 | 1.106 | (0.014,2.198) | 0.047 | -0.073 | (-0.192,0.046) | 0.230 | 0.236 | (-0.766,1.239) | 0.644 | 0.007 | (-0.029,0.043) | 0.704 | -0.036 | (-0.170,0.098) | 0.598 |
| High temperature | -0.800 | (-2.359,0.759) | 0.315 | -0.940 | (-2.068,0.187) | 0.102 | -0.004 | (-0.128,0.119) | 0.943 | -0.036 | (-1.298,0.773) | 0.619 | -0.002 | (-0.039,0.035) | 0.916 | 0.082 | (-0.057,0.220) | 0.247 |
| VDT operation | 1.167 | (-0.610,2.944) | 0.198 | 1.470 | (0.185,2.756) | 0.025 | 0.091 | (-0.049,0.232) | 0.202 | 1.093 | (-0.087,2.273) | 0.069 | -0.002 | (-0.044,0.041) | 0.944 | 0.050 | (-0.108,0.208) | 0.531 |
| Electromagnetic radiation | 1.354 | (-1.027,3.735) | 0.265 | 0.823 | (-0.899,2.545) | 0.349 | 0.086 | (-0.102,0.274) | 0.368 | 1.252 | (-0.329,2.833) | 0.121 | -0.007 | (-0.064,0.049) | 0.800 | -0.013 | (-0.225,0.198) | 0.903 |
| Ionizing radiation | -0.204 | (-2.010,1.603) | 0.825 | 0.476 | (-0.830,1.783) | 0.475 | -0.064 | (-0.207,0.079) | 0.379 | -0.557 | (-1.757,0.642) | 0.362 | 0.001 | (-0.041,0.044) | 0.948 | -0.089 | (-0.249,0.072) | 0.279 |
| Smoking | 0.235 | (-1.204,1.675) | 0.748 | 0.708 | (-0.334,1.750) | 0.183 | 0.065 | (-0.049,0.178) | 0.265 | 2.275 | (1.318,3.231) | ＜0.001 | -0.067 | (-0.101,-0.032) | ＜0.001 | 0.269 | (0.141,0.397) | ＜0.001 |
| Drinking | 2.020 | (0.699,3.340) | 0.003 | 1.473 | (0.518,2.428) | 0.003 | 0.059 | (-0.046,0.163) | 0.271 | 1.631 | (0.754,2.508) | ＜0.001 | -0.012 | (-0.043,0.019) | 0.446 | 0.116 | (-0.001,0.233) | 0.053 |
| E/R radio | -2.719 | (-6.280,0.842) | 0.134 | -1.027 | (-3.603,1.549) | 0.434 | -0.139 | (-0.421,0.142) | 0.331 | 1.189 | (-1.176,3.554) | 0.324 | -0.024 | (-0.108,0.060) | 0.578 | 0.083 | (-0.233,0.400) | 0.605 |
| Over-commitment | 0.222 | (-0.070,0.514) | 0.136 | 0.101 | (-0.110,0.313) | 0.347 | 0.030 | (0.007,0.053) | 0.011 | -0.020 | (-0.214,0.174) | 0.838 | -0.003 | (-0.010,0.004) | 0.449 | 0.013 | (-0.013,0.039) | 0.343 |

Note: Nation was “Han” as the reference group; Marital status was “Single” as the reference group; Education was “Junior college and below” as the reference group; Working system was “Regular day shift” as the reference group; Chemical toxicant, Noise, Dust, High temperature, VDT operation, Electromagnetic radiation and ionizing radiation were “No” as the reference group; Smoking and Drinking were “No” as the reference group. D/C ratio, social support, E/R ratio and over-commitment are continuous variables.

With adjustments for Nation, Marital status, Education, Work system, Smoking, Drinking and occupational hazards (Chemical toxicant, Noise, Dust, High temperature, VDT operation, Electromagnetic radiation and ionizing radiation).
